# Caloric Restriction Combined with Immobilization as Translational Model for Sarcopenia Expressing Key-Pathways of Human Pathology

**DOI:** 10.14336/AD.2022.1201

**Published:** 2023-06-01

**Authors:** Jelle C.B.C de Jong, Martien P.M Caspers, Nanda Keijzer, Nicole Worms, Joline Attema, Christa de Ruiter, Serene Lek, Arie G Nieuwenhuizen, Jaap Keijer, Aswin L Menke, Robert Kleemann, Lars Verschuren, Anita M van den Hoek

**Affiliations:** ^1^Department of Metabolic Health Research, The Netherlands Organization for Applied Scientific Research (TNO), Leiden, The Netherlands.; ^2^Human and Animal Physiology, Wageningen University, Wageningen, the Netherlands.; ^3^Department of Microbiology and Systems Biology, The Netherlands Organization for Applied Scientific Research (TNO), Zeist, The Netherlands.; ^4^Clinnovate Health UK Ltd, Glasgow, United Kingdom.

**Keywords:** Muscle atrophy, muscle-aging animal model, transcriptomics, meta-analysis

## Abstract

The prevalence of sarcopenia is increasing while it is often challenging, expensive and time-consuming to test the effectiveness of interventions against sarcopenia. Translational mouse models that adequately mimic underlying physiological pathways could accelerate research but are scarce. Here, we investigated the translational value of three potential mouse models for sarcopenia, namely partial immobilized (to mimic sedentary lifestyle), caloric restricted (CR; to mimic malnutrition) and a combination (immobilized & CR) model. C57BL/6J mice were calorically restricted (-40%) and/or one hindleg was immobilized for two weeks to induce loss of muscle mass and function. Muscle parameters were compared to those of young control (4 months) and old reference mice (21 months). Transcriptome analysis of *quadriceps* muscle was performed to identify underlying pathways and were compared with those being expressed in aged human *vastus lateralis* muscle-biopsies using a meta-analysis of five different human studies. Caloric restriction induced overall loss of lean body mass (-15%, p<0.001), whereas immobilization decreased muscle strength (-28%, p<0.001) and muscle mass of hindleg muscles specifically (on average -25%, p<0.001). The proportion of slow myofibers increased with aging in mice (+5%, p<0.05), and this was not recapitulated by the CR and/or immobilization models. The diameter of fast myofibers decreased with aging (-7%, p<0.05), and this was mimicked by all models. Transcriptome analysis revealed that the combination of CR and immobilization recapitulated more pathways characteristic for human muscle-aging (73%) than naturally aged (21 months old) mice (45%). In conclusion, the combination model exhibits loss of both muscle mass (due to CR) and function (due to immobilization) and has a remarkable similarity with pathways underlying human sarcopenia. These findings underline that external factors such as sedentary behavior and malnutrition are key elements of a translational mouse model and favor the combination model as a rapid model for testing the treatments against sarcopenia.

## INTRODUCTION

Sarcopenia is age-related loss of muscle mass and strength and is becoming a major public health problem in older people [1,2]. Sarcopenia introduces many risk factors, such as an increased risk for fractures [3], physical dependence [4] and hospitalization [5], and is associated with multiple comorbidities (e.g., diabetes mellitus type 2 and cardiovascular disease) [6-9]. This makes sarcopenia a critical driver of frailty and mortality in older people [10, 11], with a concomitant impact on healthcare costs [12]. To properly address this public health challenge, it is of paramount importance to develop interventions that can effectively prevent or reverse sarcopenia. However, it is often challenging, expensive and time-consuming to develop and test the effectiveness of such interventions in humans. It is therefore important that validated mouse models for sarcopenia are available, that can accurately predict the effectiveness of sarcopenia related interventions in humans and accelerate research within the field of sarcopenia.

Different types of mouse models have been developed and studied so far [13,14]. These include genetically modified mice, which can serve as useful models to evaluate a specific gene or pathway that is associated with sarcopenia. However, the translatability of genetically modified mice is questionable, since a single gene mutation seldom recapitulates all the underlying pathways of natural occurring sarcopenia. In addition, genetic modification may introduce exaggerated effects, which are not relevant for the pathophysiology of sarcopenia. Old mice are often considered to be translational, however, the use of old mice comes with several challenges, as it can be time-consuming and expensive to use them. In addition, aging introduces aging-related diseases, which often remain unnoticed and can make the respective results difficult to interpret. Consequently, new animal models are required that preferably display a broad spectrum of biological processes involved in sarcopenia, whilst preferably also being easy to use and time- and cost-effective.

Therefore, our main objective was to test the translatability of three novel mouse models, in which sarcopenia was modelled using caloric restriction (CR) to mimic malnutrition, immobilization to mimic a sedentary lifestyle, or a combination thereof (CR and immobilization). Malnutrition is an important risk factor for the incidence for sarcopenia, as adequate nutritional intake is essential for maintaining muscle mass [15-18] and especially older people are susceptible for malnutrition induced muscle atrophy [19]. The correlation of malnutrition with sarcopenia might be due to a causal relationship as it has been postulated that malnutrition (de-)activates pathways underlying the pathophysiology of sarcopenia [19]. In addition, a large meta-analysis found that the prevalence of malnutrition is relatively high in European older people [20], making malnutrition not only a physiologically potent driver of sarcopenia, but also societally relevant. Furthermore, a sedentary lifestyle has been found to be a significant risk factor for poor muscle function in older people as well [21] and is suspected to be an important factor in the pathogenesis of sarcopenia [22,23]. Based on these observations we hypothesize that the combination of CR and immobilization could be used to generate a translatable mouse model that is cost and time effective as well. We analyzed muscle mass, function and histology of these novel mouse models and compared them with aged mice. In addition, we assessed the translational value of the models by muscle gene expression profiling and comparison to human sarcopenia.

## MATERIALS AND METHODS

### Animals and experimental design

All animal care and experimental procedures were approved by the Ethical Committee on Animal Care and Experimentation (Zeist, The Netherlands; approval reference numbers TNO-436, date: 17^th^ of September 2019, and TNO-440, date: 24^th^ of October 2019), and were in compliance with European Community specification regarding the use of laboratory animals.

Ten-week-old male C57BL/6J mice were obtained from Charles River Laboratories (L’Arbresle, France) and were kept on chow maintenance diet (Ssniff Spezialdieten GmbH, Soest, Germany). Male mice were used since those are heavier and contain more muscle mass compared to female mice. Mice were housed individually in a temperature-controlled room on a 12h light-dark cycle and had free access to food (unless mentioned otherwise) and water. At the age of 12 weeks, *ad libitum* caloric intake was determined for each mouse separately by measuring normal individual baseline caloric intake over three periods of 3 days. At the age of 14 weeks, mice were matched for body weight and lean body mass and sub-divided into four groups. One control group (n=12) remained on *ad libitum* chow, one CR group (n=12) received 60% of the kcal (chow) normally consumed under *ad libitum* conditions, one immobilized group (n=12) remained on *ad libitum* chow, but right hindleg was immobilized, and one combination (CR + immobilization) group (n=12) received 60% of the kcal (chow) normally consumed under *ad libitum* conditions and their right hindleg was immobilized as well. This period of caloric restriction (CR) and/or immobilization lasted for 14 days. Immobilization was achieved through fixation of the right hind leg using adhesive bandage and surgical tape and resulted in a fixation of the knee joint at 180° [24]. The adhesive bandage and surgical tape were checked daily and replaced on day 5 and day 10 of immobilization, and on any other day if the bandage became loose. Body weight and lean body mass were measured on day 0, 3, 7, 10 and 13 of CR and/or immobilization using an NMR EchoMRI 2-in-1 whole body composition analyzer (Echo Medical Systems LTD, Houston, TX, USA) [25]. Grip strength of fore- and hind paws was measured on day 13 using a Grip Strength Meter (TSE Systems, Bad Homburg, Germany). Five trials were performed, the trial with highest and lowest force were excluded and the maximal force of the three remaining trials was averaged [26]. Furthermore, voluntary movement of the mice (at least 8 per group) was measured during the second week of CR and/or immobilization. Average speed and travelled distance were measured every 30 minutes by means of infrared sensors using TSE PhenoMaster V4.6.2 (TSE Systems, Bad Homburg, Germany) during two consecutive days and nights. Data of the second day and night was used. Finally, after 14 days, mice were sacrificed by CO_2_ inhalation and hind limb muscles were dissected and weighted. These mice were 16 weeks old at this timepoint. *Gastrocnemius* and *soleus* muscles were formalin-fixed and paraffin-embedded for histological analysis, while *quadriceps* muscles were frozen in liquid nitrogen for transcriptome analysis.

For reference, naturally aged mice were used. To do so, 13 months (58 weeks) old male C57BL/6J mice were obtained from Charles River Laboratories and were kept on chow maintenance diet (Ssniff Spezialdieten GmbH, Soest, Germany). Mice were housed individually in a temperature-controlled room on a 12h light-dark cycle and had free access to food and water. At 17 months of age (74 weeks), all mice were matched for body weight and lean body mass and subdivided into two groups: one 17 months (76 weeks) and one 21 months (92 weeks) old group. Grip strength, voluntary movement and body composition of these aged mice were performed after 17 or 21 months similarly as described for the other models and mice were sacrificed thereafter to collect tissues representing the natural aging process. The naturally aging mice were aged until 17-21 months, as various sarcopenia related muscle parameters (e.g. muscle mass) have been reported to be impaired at those timepoints [27,28].

### Histological analysis

*Gastrocnemius* and *soleus* muscles were isolated jointly, fixed in formalin, cross-sectionally cut at their thickest part and embedded in paraffin. Cross-sections of 5 µm were cut and slow (type 1 myofibers) and fast (type 2 myofibers) myosin heavy chains were stained. Slides were deparaffinized and epitope was retrieved through a 25-minute incubation in 0.1 mM EDTA at 95°C, followed by a 5-minute incubation in 0.1% trypsin(v/v) and 0.1% calcium chloride (w/v) at 37°C. Endogenous peroxidase activity was masked by a 10-minute incubation in 0.3% hydrogen peroxide (v/v) and slides were blocked by a 10 minutes incubation in 20% normal rabbit serum in TBS. After blocking, slow myosin heavy chains were immunolabeled by a 30-minute incubation in 1/2000 mouse monoclonal anti-slow myosin antibody (SAB4200670; Sigma-Aldrich), followed by a 1-hour incubation in 1/50 polyclonal rabbit anti-mouse HRP (P0260; Dako) and ultimately developed using an HRP substrate kit (SK-4700; Vector). Fast myosin heavy chains were immunolabeled by a 1-hour incubation in 1/50 alkaline phosphatase conjugated mouse anti-fast myosin antibody (A4335; Sigma-Aldrich) and developed using a red alkaline phosphatase substrate kit (SK5100; Vector). For negative controls, the incubation step with the primary antibody was omitted. After development, the slides were dehydrated and covered. Myofiber diameter was quantified by measuring minimal Feret’s diameters using ImageJ, since this parameter is least sensitive to sectioning angle [29]. This analysis was performed in 5 randomly selected regions of interest (0.18 mm^2^) in 1-2 cross sections per animal. In s*oleus* muscle this was performed for each myofiber type separately. In two immobilized mice myofiber size of type 1 myofibers in the *soleus* muscle was not measured since they only had type 2 myofibers. In *gastrocnemius* muscle we did not discriminate between slow and fast myofibers since the *gastrocnemius* muscle in mice consists of primarily type 2 (fast) myofibers [30].

In addition, collagen was quantified as extracellular matrix collagen deposition has previously been reported to increase with aging in muscle, and possibly contributes to stiffening of muscle [31]. To do so, paraffin-embedded unstained sections of *gastrocnemius* and *soleus* muscles were used for Second Harmonic Generation (SHG) imaging of collagen using a Genesis®200 imaging system and subsequent computer-assisted data analysis (HistoIndex, Singapore). SHG is a non-linear optical process highly sensitive to non-centrosymmetric structures such as collagen fibrils and fibers [32]. Collagen was quantified by measuring mean collagen fiber thickness and length. In addition, 5 µm cross-sections were stained with Sirius Red and muscular fibrosis was identified using computerized image analysis of muscle collagen content (as percentage of muscle surface area).

### Transcriptome analysis

RNA extraction was performed as described previously in detail [33]. Total RNA was extracted from individual *quadriceps* muscle samples using glass beads and RNA-Bee (Campro Scientific, Veenendaal, The Netherlands). RNA integrity was examined using the RNA 6000 nano Lab-on-a-Chip kit and a bioanalyzer 2100 (Agilent Technologies, Amstelveen, The Netherlands). The NEBNext Ultra II Directional RNA Library Prep Kit (NEB #E7760S/L, New England Biolabs, Ipswich, MA, USA) was used to process the samples. Briefly, mRNA was isolated from total RNA using the oligo-dT magnetic beads. After fragmentation of the mRNA, cDNA synthesis was performed, and cDNA was ligated with the sequencing adapters and amplified by PCR. The quality and yield of the amplicon were measured (Fragment Analyzer, Agilent Technologies, Amstelveen, The Netherlands). The size of the resulting product was consistent with the expected size distribution (a broad peak between 300-500 bp). Clustering and DNA sequencing, using the Illumina NovaSeq6000, was performed according to manufacturer's protocols of service provider GenomeScan B.V. (Leiden, the Netherlands) using a concentration of 1.1 nM of amplicon library DNA and yielding at least 15 million sequencing clusters per sample and 150nt paired-end reads. The genome reference and annotation file Mus_musculus. GRCm38.gencode.vM19 was used for analysis in FastA and GTF format. The reads were aligned to the reference sequence using the STAR 2.5 algorithm with default settings (https://github.com/alexdobin/STAR). Based on the mapped read locations and the gene annotation, HTSeq-count version 0.6.1p1 was used to count how often a read was mapped on the transcript region. These counts served as input for the statistical analysis using the DEseq2 package [34].

### Meta-analysis

To assess the recapitulation of pathways associated to human muscle aging in the mouse models, we compared differentially expressed genes and pathways of our model vs. young control to the differentially expressed genes and pathways of old humans vs. young controls. Gene expression profiles of old and young human *vastus lateralis* muscle biopsies were aggregated from five different human studies (Table 1). Gene expression profiles of old (n=172) versus young (n=138) human participants were compared. In the case of intervention studies, profiles of baseline measurements were used. Genes were considered to be differentially expressed, if a gene was differentially expressed in the same direction in two or more studies. Differentially expressed genes (DEGs) were used as an input for pathway analysis (p-value<0.01) through Ingenuity Pathway Analysis suite (www.ingenuity.com, accessed 2021).

### Statistical analysis

Data analysis (of data other than transcriptomics data) was performed using SPSS Statistics 27 (IBM, Armonk, NY, USA). No mice or data points were excluded during analysis. Normality of data was tested using the Shapiro-Wilk and Kolmogorov-Smirnov test. If data was normally distributed, then a two-way ANOVA was used follow by a Tukey’s test for post-hoc analysis. If data was not normally distributed, then a Kruskal-Wallis test was used followed by Mann-Whitney U test for independent samples. A p-value of <0.05 was considered statistically significant, two-tailed p-values were used and all values are displayed as mean ± SEM.

**Table 1:** Overview of included GEO (gene expression omnibus) datasets for meta-analysis.

Study	Number of participants		Age range (yrs: min-max)		
	Young	Old	Young	Old	Muscle
GSE144304 [53]	26	54	20-27	75-90	*Vastus lateralis*
GSE157585 [54]	21	30	20-30	65-91	*Vastus lateralis*
GSE117525 [55]	51	47	18-29	65-96	*Vastus lateralis*
GSE8479 [56]	26	25	18-28	65-84	*Vastus lateralis*
GSE362&GSE674 [57,58]	14	16	20-29	65-75	*Vastus lateralis*
Total	138	172	18-30	≥65	

## RESULTS

### Caloric restriction induces loss of lean body mass and immobilization loss of muscle strength

**Figure 1.**
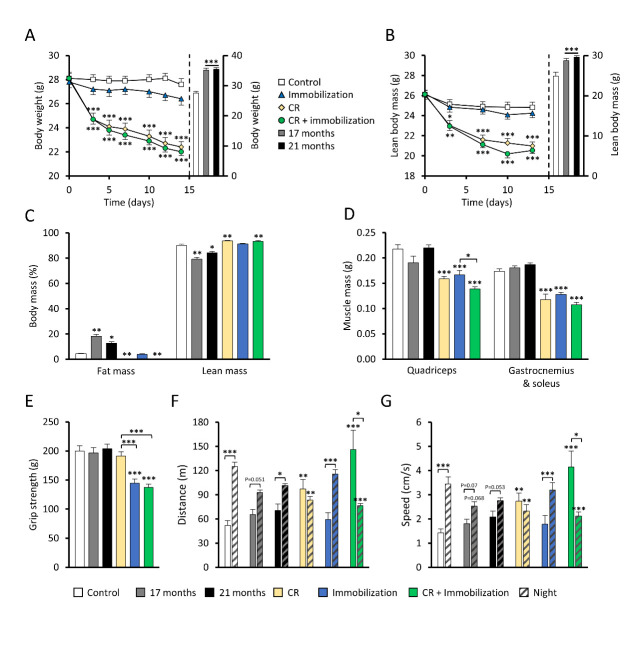
Body composition and functional measurements of mouse models (all groups n=12 unless mentioned otherwise). (A) Body weight over-time of 4 months old mice exposed for 14 days to caloric restriction (CR), immobilization, combination of CR and immobilized mice, and control mice. The body weight of the aged mice in bars (right) is their body weight in week of sacrifice. (B) Total lean body mass over-time in the control, CR, immobilization, or combination mice. The total lean body mass of aged mice in bars (right) is their total lean body mass in week of sacrifice. (C) Relative fat and lean mass (%) in week of sacrifice. (D) Wet muscle weight directly after sacrifice. (E) Grip strength of all mouse models shortly before sacrificing. (F) Average voluntary travelled distance (per hour) or average speed (G) of 17 months old mice (n=8), 21 months old mice (n=12), mice exposed to CR and/or immobilization (n=12) or control mice (n=12) during day or night-time. In graphs A, B, C, F and G data was not normally distributed and a Kruskal-Wallis test followed by a Mann-Whitney U test was used. In all other graphs data was normally distributed and a two-way ANOVA followed by a Tukey’s test for post-hoc analysis was used. Values represent mean ± SEM. *p<0.05, **p<0.01 and ***p<0.001 vs. control mice unless indicated differently.

As expected, mice that were calorically restricted (with -40%) for 14 days lost 18% total body weight and 15% of lean mass in comparison to *ad libitum* fed control mice (both p<0.001, Fig. 1A-B). Immobilization did not have a significant effect on body weight or lean body mass (Fig. 1A-B), nor on food intake (Suppl. Fig. 1). Mice exposed to the combination of CR and immobilization lost 20% of their total body weight and 17% of their lean body mass compared to control mice (both p<0.001) which was comparable to CR alone. Contrastingly, naturally aged mice (both 17- and 21-months old mice) showed increased body weight and lean body mass as compared to young (4 months old) control mice (both p<0.001).

Analysis of body composition revealed that in naturally aged mice relative lean mass was decreased in both 17- and 21-months old mice compared to young control mice (-10.7%, p<0.01 and -5.6%, p<0.05 respectively; Fig. 1C). Furthermore, mice exposed to CR, or the combination of CR and immobilization significantly lost relative fat mass (both p<0.01) and slightly increased in relative lean body mass, while body composition of immobilized mice did not change.

Weight of hindleg muscles at sacrifice revealed that in mice exposed to CR or immobilization, weight of *quadriceps* (-27% and -23%, respectively, both p<0.001) and *gastrocnemius* + *soleus* muscles (-32% and -26%, respectively, both p<0.001, Fig. 1D) were significantly decreased, compared to control mice. This was exceeded by mice exposed to both CR and immobilization, which lost 35% weight of their *quadriceps* muscles and 37% of their *gastrocnemius* + *soleus* muscles (both p<0.001). On the contrary, weight of hindleg muscles of naturally aged mice did not change compared to control mice.

**Figure 2.**
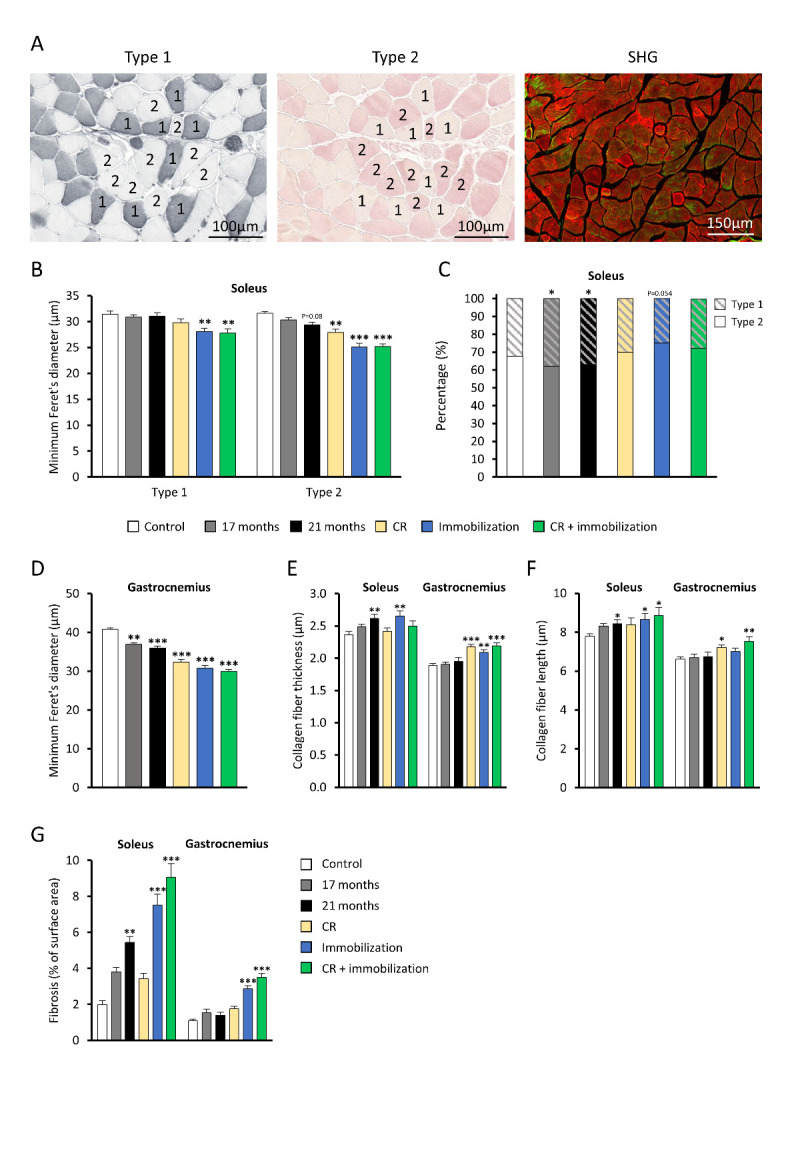
Histological measurements in *soleus* and *gastrocnemius* muscles. Groups include 17 months old (n=12) and 21 months old (n=11) mice, and 4 months old mice exposed for 14 days to caloric restriction (n=12), immobilization (n=12 and n=10 for type 1 myofiber diameter measurements), combination of caloric restriction and immobilization (n=11) and control mice (n=11), unless mentioned otherwise. (A) Representative images of *soleus* muscle stained with immunohistochemical staining of slow (type 1) and fast (type 2) myosin heavy chains and Second Harmonic Generation (SHG) imaging of collagen (green signal). (B) Minimum Feret’s diameter of *soleus* muscle of both type 1 and type 2 myofibers. (C) Percentage of type 1 and type 2 myofibers in *soleus* muscle within each group. (D) Minimum Feret’s diameter of myofibers in *gastrocnemius* muscle. (E) Collagen fiber thickness and (F) length in *soleus* and *gastrocnemius* muscle in 17 months old (n=12) and 21 months old mice (n=12), and 4 months old mice exposed to caloric restriction (n=11), immobilization (n=12), combination of caloric restriction and immobilization (n=11) and control mice (n=12). (G) Fibrosis in *soleus* and *gastrocnemius* muscle indicated by percentage of positive Sirius Red area. In graphs C, E and F data was not normally distributed and a Kruskal-Wallis test followed by a Mann-Whitney U test was used. In all other graphs data was normally distributed and a two-away ANOVA followed by a Tukey’s test for post-hoc analysis was used. Values represent mean ± SEM. *p<0.05, **p<0.01 and ***p<0.001 vs. control mice.

**Figure 3.**
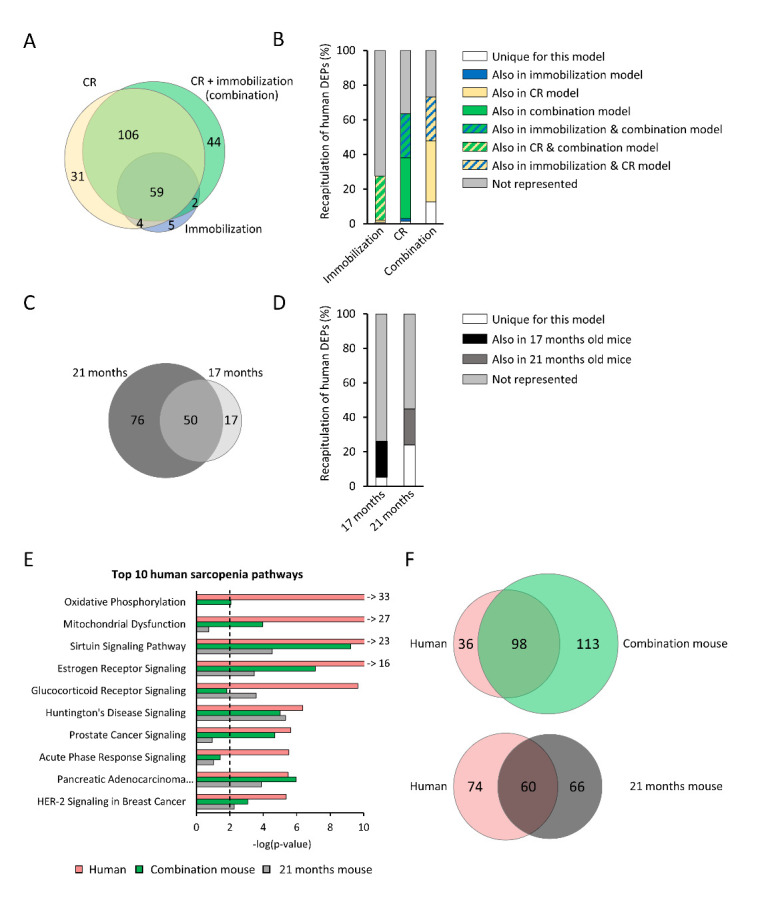
Transcriptome analysis of quadriceps muscle of 4 months old mice exposed for 14 days to caloric restriction, to immobilization, to a combination of caloric restriction (CR) and immobilization, a control group, and 17- and 21-months old mice (all groups n=12). (A) Venn-diagram of unique and overlapping differentially expressed pathways (DEPs) for CR, immobilized and combination of CR and immobilization vs. control mice. (B) Visualization of DEPs underlying human muscle-aging and representation thereof in the different models. In total, 134 DEPs associated to human muscle-aging were found using five different human studies (old vs. young human participants) and set as 100%. The percentage of human DEPs that were recapitulated by the mouse models (vs. control mice) are displayed. The color coding indicates whether a DEP that overlapped with human-muscle aging was unique for the respective model, shared with one other model or shared with all three models. (C) Venn-diagram of unique and overlapping DEPs for 17 months old and 21 months old mice (vs. control mice). (D) The 134 DEPs associated to human muscle-aging were set as 100% and the percentage of DEPs overlapping with human muscle-aging are indicated as either unique for the respective model or shared with the other aged model. (E) Top 10 DEPs of human muscle-aging and the differential expression (-log(p-value)) of the combination and 21 months models for the same pathways. Arrows with numbers indicate -log(p-value) of pathways with -log(p-value) greater than 10. (F) Venn-diagrams indicating the number of overlapping DEPs of the combination and 92 months model with human muscle-aging DEPs.

Measurement of functional muscle parameters revealed that naturally aged mice did not lose grip strength as compared to young control mice (Fig. 1E). Calorically restricted mice did not lose grip strength as well, while immobilized mice and immobilized mice with caloric restriction did lose grip strength (-17% and -21%, respectively, both p<0.001), indicating that immobilization is necessary to impact grip strength. Analysis of voluntary movement revealed a clear day-night pattern in young control mice, with higher activity (both distance and speed) during the night, and this pattern seemed less prominent in naturally aged mice (Fig. 1F-G). The average movement speed of mice during the night tended to decrease with aging at 17 months (p = 0.068; Fig. 1F-G), but the difference did not become significant at 21 months compared to the young control group, and average distance was not significantly changed. Caloric restriction led to a higher activity (both distance and speed) during daytime and lower activity during nighttime as compared to the young control group. Remarkably, this effect was even more prominent in the combination group, while immobilization itself did not have an effect on voluntary movement (both distance and speed).

**Figure 4.**
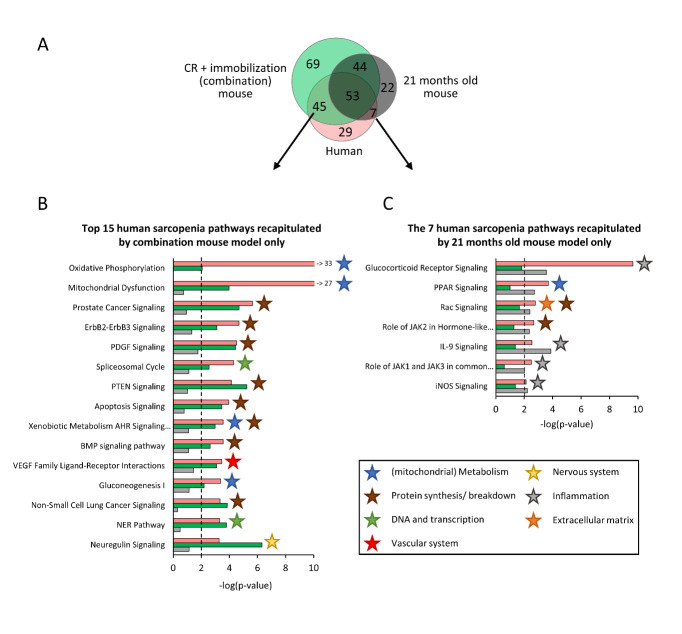
Comparison of recapitulation of DEPs associated to human muscle aging in either mice exposed for 14 days to the combination of caloric restriction (CR) and immobilization (n=12) or to aging for 21 months (n=12). (A) Venn-diagram displaying the overlap between DEPs of human muscle-aging, combination mouse model of CR and immobilization, and the 21 months old mouse model. (B) Top 15 DEPs that are recapitulated by the combination mouse model but not by the aged mouse model. (C) The seven DEPs that are recapitulated by the aged mouse model but not by the combination mouse model. Stars indicate in which biological category a pathway was classified.

### Novel mouse models display loss of type 1 and type 2 myofibers

Diameter of type 1 (slow) myofibers were histologically analyzed (Fig. 2A) and were smaller in immobilized and combination mice (both with -11%, both p<0.01, Fig. 2B). No differences were detected in naturally aged mice and mice exposed to CR, altogether indicating that immobilization is necessary to significantly induce type I myofiber atrophy. Atrophy of type 2 (fast) myofiber was observed in CR (-12%, p<0.01), immobilized (-21%, p<0.001) and combination mice (-20%, p<0.001). The diameter of type 2 myofibers tended to decrease slightly during natural aging and was 7% smaller in 21 months old mice compared to young control mice (p = 0.08). Furthermore, in CR and combination mice no statistically significant shift in myofiber type distribution was observed, while in immobilized mice a tendency for a shift towards type 2 myofibers was observed (p = 0.054, Fig. 2C). In both 17 months (+17%) and 21 months (+14%) old mice the proportion of type 1 myofibers increased (both p<0.05).

In *gastrocnemius* muscle, we did not discriminate between slow and fast myofibers since the *gastrocnemius* muscle in mice consists of primarily type 2 (fast) myofibers [30]. The average *gastrocnemius* myofiber diameter was decreased in CR (-21%), immobilized (-25%) and combination (-27%) mice (all p<0.001, Fig. 2D). This atrophic effect also occurred during natural aging and *gastrocnemius* myofiber diameter was 10% smaller in 17 months old mice (p<0.01) and 12% smaller in 21 months old mice (p<0.001) compared to control mice. Distribution frequency graphs of myofiber diameter are added as supplementary figure (Supplementary Fig. 2A-C).

Extracellular matrix collagen deposition has previously been reported to increase with aging in muscle, and possibly contributes to stiffening of muscle [31]. In the current study, collagen content was quantified by measuring collagen fiber thickness and length using Second Harmonic Generation (Fig. 2A, right panel). Collagen fiber thickness was increased in *soleus* muscle of 21 months old mice vs. young control mice (+11%, p<0.01, Fig. 2E) and this was recapitulated in immobilized mice (+12%, p<0.01), but not by CR or in the combination mouse model, nor in 17 months old mice (Fig. 2E). In *gastrocnemius* muscle, collagen fiber thickness did not increase with aging in 17- and 21-months old mice, but did increase after immobilization (+15%, p<0.01), CR (+11, p<0.001) and in the combination model (+16%, p<0.001). Furthermore, length of collagen fibers increased in CR and combination mice in *gastrocnemius* muscle (p<0.05 and p<0.001, respectively, Fig. 2F) and was increased in 21-months old mice, immobilized and combination mouse models in *soleus* muscle as well (all p<0.05). Sirius Red staining was used to quantify fibrotic area (Fig. 2G), and in *soleus* muscle this was increased in 21 months old mice (p<0.01) and in immobilized and combination mouse models as well (p<0.001). In *gastrocnemius* muscle fibrosis increased in immobilized and combination mouse models (p<0.001) and did not increase with aging in contrast to the *soleus* muscle, consistent to the SHG data.

### The combination model recapitulates more pathways associated to human sarcopenia than the aged model

To gain insight in the pathways underlying muscle-atrophy in the models, transcriptome analysis was performed in *quadriceps* muscles, and gene expression profiles of the different mouse models were compared to the gene expression profiles of control mice. Immobilization induced a total of 70 differentially expressed pathways (DEPs), while CR and the combination of CR and immobilization induced many more DEPs (n=200 and n=211, respectively, Fig. 3A). Immobilization or CR *per se* induced some unique pathways (n=5 and n=31, respectively), but the majority of the DEPs of these models were shared with the combination model (n=167). In addition, the combination of CR and immobilization introduced 44 DEPs that were unique for this model.

To investigate the translational value of our models for human sarcopenia, we determined how many of the pathways that characterize human sarcopenia were differentially expressed in our mouse models as well. To this end, first a meta-analysis was performed, in which transcriptomics data of five different human studies were aggregated (Table 1). In total, 134 DEPs were differentially expressed in old vs. young human skeletal muscle. Immobilized mice recapitulated 28% of these human pathways, and the percentage of recapitulated human DEPs increased to 63% in the CR mice (Fig. 3B). The combination mice recapitulated the largest percentage of human DEPs, namely 73%, which substantially overlapped with the pathways that were recapitulated by the CR mice or immobilized mice as well. Interestingly, the combination of CR and immobilization introduced many human DEPs that were uniquely represented in this model, including noticeable pathways related to (mitochondrial) metabolism (e.g., ‘oxidative phosphorylation’ and ‘gluconeogenesis I’). Moreover, similar analyses were performed for 17 months and 21 months old naturally aged mice (Fig. 3C). At 17 months, aging introduced a total of 67 DEPs, and at 21 months this increased to 126 DEPs. However, the percentage of human DEPs that were recapitulated was relatively low compared to the combination model. In the 17 months old mice the percentage of human DEPs that were recapitulated was 26%, and in the 21 months old mice this recapitulation increased to 45% (Fig. 3D).

Since the combination model recapitulated most of the DEPs that were found in humans, we compared this model with the 21 months old aged model in more detail. We examined the top 10 pathways for human sarcopenia and found that 8 out of 10 DEPs were recapitulated by the combination model, whilst 6 out of 10 were differentially expressed in the 21 months old mice (Fig. 3E). Noticeably, the two highest ranked human pathways, ‘oxidative phosphorylation’ and ‘mitochondrial dysfunction’, were not recapitulated by the old mice. Venn-diagrams confirmed that the combination model recapitulated more pathways associated to human-muscle aging than the model of naturally aging, and also showed that both models differentially express pathways that did not overlap with human muscle-aging DEPs (Fig. 3F). More information on these DEPs can be found in supplementary Figure 3.

Furthermore, we compared which human DEPs were uniquely recapitulated by the CR and immobilization combination model, and which human DEPs were uniquely recapitulated by the 21 months old mice. We identified 45 human DEPs that were differentially expressed in the combination model, but not in the naturally aged mice (Fig. 4A), which comprised predominantly pathways related to protein synthesis/breakdown, (mitochondrial) metabolism and DNA and transcription (Fig. 4B). By contrast, the aging model recapitulated only seven human DEPs that were not recapitulated by the combination model, and these were mainly related to inflammation.

**Figure 5.**
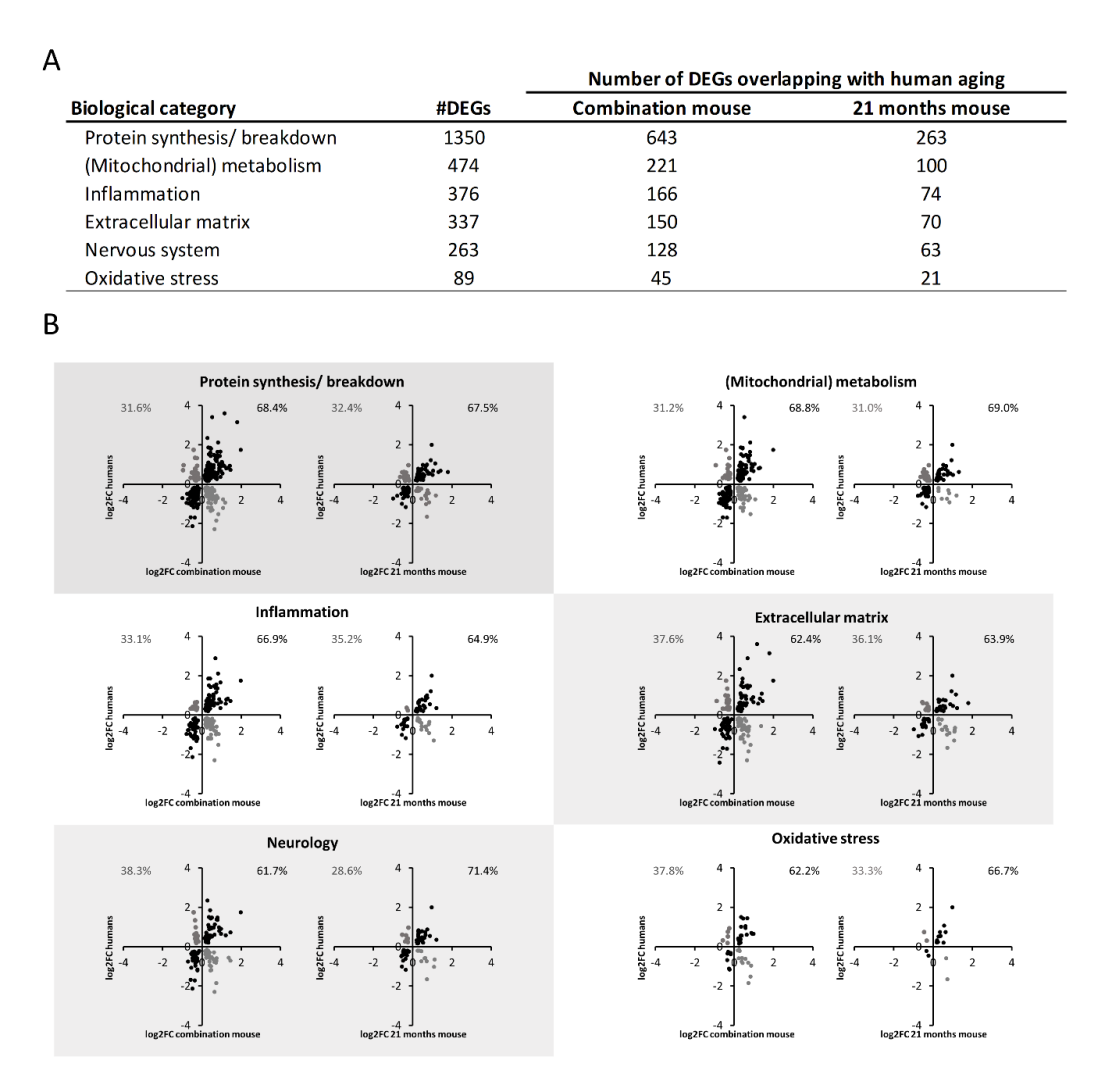
Analysis of recapitulation by mouse models of transcriptional features associated to human muscle aging on gene level. (A) All human DEPs were classified in a biological category, and for each biological category it was calculated how many of the underlying DEGs were also differentially expressed in mice exposed for 14 days to the combination of caloric restriction and immobilization and in the 21 months old mice. (B) Correlation plots displaying whether the direction of regulation of an overlapping DEG was similar in old humans vs. young controls (x-axis) and the mouse models vs. controls (y-axis). Percentages in the left upper corner (grey) display the percentage of shared DEGs in the first or fourth quartile of the correlation plot (which indicates DEGs regulated in opposite direction). Percentages in upper right corner (black) display the percentage of shared DEGs in second or third quartile of the correlation plot (which indicates DEGs regulated in similar direction).

### Combination model also recapitulates direction of regulation of DEGs underlying human sarcopenia

Besides significant regulation on pathway level, it is important to investigate whether the direction of differential expression of underlying genes was recapitulated by the models as well. To do so, we categorized human DEPs in biological categories (see supplementary materials) and determined for each biological category how many of the underlying DEGs were recapitulated by the combination and 21 months old model, and how many of these recapitulated DEGs displayed a similar direction of regulation. In each biological category more human DEGs were differentially expressed by the combination mouse model than by the aged mouse model, as also suggested by the pathways analysis. More importantly, the percentage of these recapitulated DEGs that were regulated in similar direction as in human sarcopenia, was often similar or higher in the combination model as compared to the naturally aging model. For example, by comparing old vs. young humans, we found 1350 DEGs involved in protein synthesis/breakdown. In the combination model 643 of these DEGs were recapitulated (Fig. 5A), and 68.4% of those DEGs were regulated in similar direction by the combination model (vs. control mice), essentially as in old humans (vs. young controls, Fig. 5B). In the same biological category, the aged mouse recapitulated less (263) DEGs, and 67.5% of these DEGs were regulated in similar direction by the old mice (vs. control mice), as in old humans (vs. young controls). Similar patterns were observed in the other biological categories, including (mitochondrial) metabolism, inflammation, extracellular matrix, neurology and oxidative stress (Fig. 5B).

## DISCUSSION

The aim of this study was to investigate the translatability of novel mouse models for muscle-aging on three different levels, namely functional, histological, and transcriptional level. We demonstrated that CR is an effective method to induce loss of (lean) body mass, and partial immobilization to induce loss of muscle strength, and that these interventions can successfully be combined in one model. Contrastingly, aging *per se* did not induce loss of muscle mass or muscle strength, although voluntary movement did tend to decrease with aging. The combination model (CR + immobilization) displayed atrophy of both type 1 and 2 myofibers, and importantly, transcriptome analysis revealed a high level of overlap with pathways underlying muscle-aging in humans (73%), which was higher in the combination model compared to 21 months old mice (45%). This is an important observation, as this suggests that immobilization and malnutrition are key strategies to create a translational mouse model for human aging, and that aging *per se* (until 21 months of age) in mice is not as sufficient. To our knowledge these results are unique, as we thoroughly assessed the translatability of both the phenotype as the underlying pathways of our models.

One of our most striking findings is that the number of pathways that were recapitulated by the combination model (73%) was higher than the number of pathways recapitulated by the aged mice (45%). The etiology of sarcopenia includes both external factors, such as malnutrition and a sedentary lifestyle, and internal factors such as a decreased oxidative metabolism, chronic low-grade inflammation, denervation, and changes in the matrisome [35]. It is frequently expected that the aging mouse model is more translatable than models that have not naturally aged, since these supposedly lack the recapitulation of these internal age-related factors. However, this proposition is strongly challenged by our findings, as we found that the transcriptional signature that is induced by aging *per se* is not more, but actually less translational than the transcriptional signature that is induced by malnutrition and immobilization. This is not an illogical finding, since strong associations between malnutrition and a sedentary lifestyle and (muscle-)aging have been found in many human studies [15-19,36-38]. For example, older adults are especially vulnerable for a sedentary lifestyle, since muscle-atrophy is higher in older adults compared to younger adults after exposure to the same sedentary period [39]. Moreover, our results indicate that the human muscle aging-trajectory is perhaps not as well conserved in mice. For example, aging did not induce loss of muscle mass and strength in the oldest group (21 months). This age in mice is estimated to correlate with 70+ human years [40], however, in humans’ loss of muscle mass and strength already becomes apparent after 50 years [41], which is a notable difference. Perhaps an even older age is required for mice to start showing functional signs of sarcopenia. Furthermore, some studies report a decreased grip strength in aged mice at 18 months or older [27,42]. However, in these studies grip strength is normalized for body weight, and body weight can increase independent of absolute loss of grip strength. Therefore, we cannot conclude whether in those studies also loss of absolute grip strength occurred, which we did not observe in the current study. Besides lack of significant aging-related loss of muscle mass and strength in mice, other factors could explain the discrepancy between human and mouse muscle-aging. Interestingly, one other study has shown that some of the internal factors of human muscle-aging are not well conserved in mice. It was found that aging-associated changes in the regulation of inflammation, immune processes and cell cycle in human skeletal muscle were not necessarily recapitulated by aged mice, which is in line with our findings [43].

Furthermore, in this study we show that muscle-disuse is required in addition to CR to induce loss of muscle strength. In both mice and humans, it has been shown that muscle-disuse leads to loss of absolute muscle strength [44-46]. Interestingly, the negative effects of muscle disuse are exacerbated in old participants compared to young participants [44,47,48], which could be explained by decreased capacity for tissue regeneration, increased inflammation or decreased neuronal motor function in older participants [44,47]. These are processes that are frequently found in studies on muscle-aging as well [35,49], showing the parallels between muscle-disuse and aging, as found in the current study. Moreover, increased collagen content possibly contributes to muscle stiffening, and we found here that muscle-disuse in immobilized mice increased collagen fiber thickness and muscle fibrosis and suggests that increased collagen deposition could be one of the mechanisms underlying immobilization induced loss of muscle function. An increased collagen deposition and muscle fibrosis was observed in old mice as well in *soleus* but not in *gastrocnemius* muscle [31]. This is an interesting finding, since the *soleus* has a more oxidative phenotype compared to *gastrocnemius* muscle, suggesting that perhaps oxidative stress plays an important role in the observed aging-related collagen deposition, but this has to be studied in greater detail.

The findings of the current study are highly relevant, as the combination model of CR and partial-immobilization offers possibilities for nutritional or pharmacological interventions and can accelerate pre-clinical research. Previous studies have already demonstrated the potential translational value of mouse models based on partial-immobilization or CR, including studies on vitamin D or Losartan [50,51]. In addition, in one of our previous studies, we used the CR mouse model and found that a novel oral nutritional supplement (Vital01) can alleviate muscle-atrophy during CR and also improve recovery post CR [25]. Later, these results were largely replicated in old humans, as Vital01 supplementation improved muscle function compared to a standard supplement [52]. The results from the current study now provide novel evidence for the translatability of mouse models based on CR and immobilization, in addition to these previous studies demonstrating the translational potential of these models. In future research, this model can be used to investigate the effects of pharmacological or nutritional interventions to attenuate or reverse muscle-atrophy related to sarcopenia or accelerate recovery after a catabolic event. Validation of the results on protein level might provide additional insights and should be included in future studies as well.

We conclude that CR is an effective way to induce muscle atrophy, and immobilization is required to induce loss of muscle strength. The combination model exhibited loss of both muscle mass and function, reduction of type 1 and 2 myofiber diameter, and illustrated substantial similarity with pathways underlying human muscle-aging. Therefore, the combination model could serve as a more cost and time-effective alternative for the aged mouse model, whilst maintaining a high degree of translatability to human sarcopenia. We conclude that the combination model can be a suitable model for testing the effectiveness of novel muscle-aging related interventions.

## Supplementary Materials

The Supplementary data can be found online at: www.aginganddisease.org/EN/10.14336/AD.2022.1201.
