# Supplementary material for: Caloric Restriction Combined with Immobilization as Translational Model for Sarcopenia Expressing Key-Pathways of Human Pathology
Source: Aging Dis. 2023 Jun 1;14(3):937–51. doi: 10.14336/AD.2022.1201 (PMC10187708; doi:10.14336/AD.2022.1201)
Supplement: Supplementary file 1 — The Supplementary data can be found online at: www.aginganddisease.org/EN/10.14336/AD.2022.1201. [file AD-14-3-937-s.pdf]

SUPPLEMENTARY DATA

Caloric Restriction Combined with Immobilization as Translational Model for Sarcopenia Expressing Key-Pathways of Human Pathology

Jelle CBC. de Jong[1,2*], Martien P.M. Caspers[3], Nanda Keijzer[1], Nicole Worms[1], Joline Attema[1], Christa de Ruiter[1], Serene Lek[4], Arie G. Nieuwenhuizen[2], Jaap Keijer[2], Aswin L. Menke[1], Robert Kleemann[1], Lars Verschuren[3], Anita M. van den Hoek[1]

© 2022. de Jong J. et al. Published online at http://www.aginganddisease.org/EN/10.14336/AD.2022.1201

SUPPLEMENTARY DATA

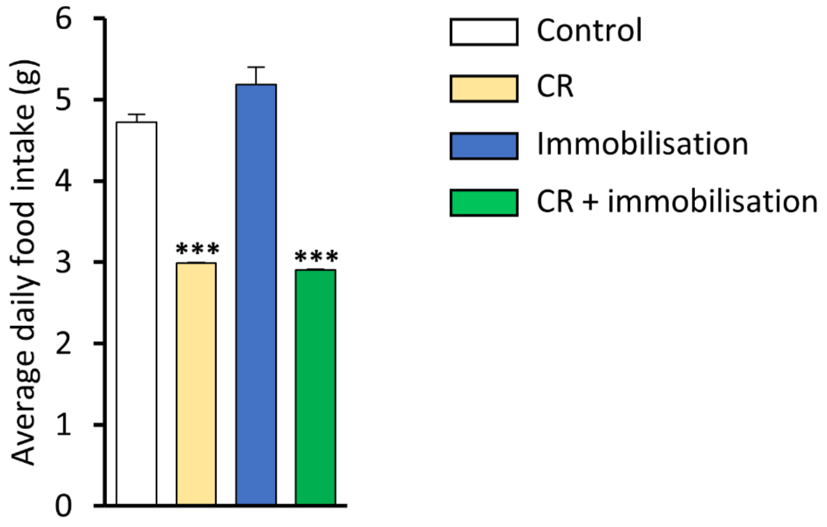

Supplementary Figure 1. Average food intake of control, calorically restricted, immobilized and combination mice during the 14-days of the study.

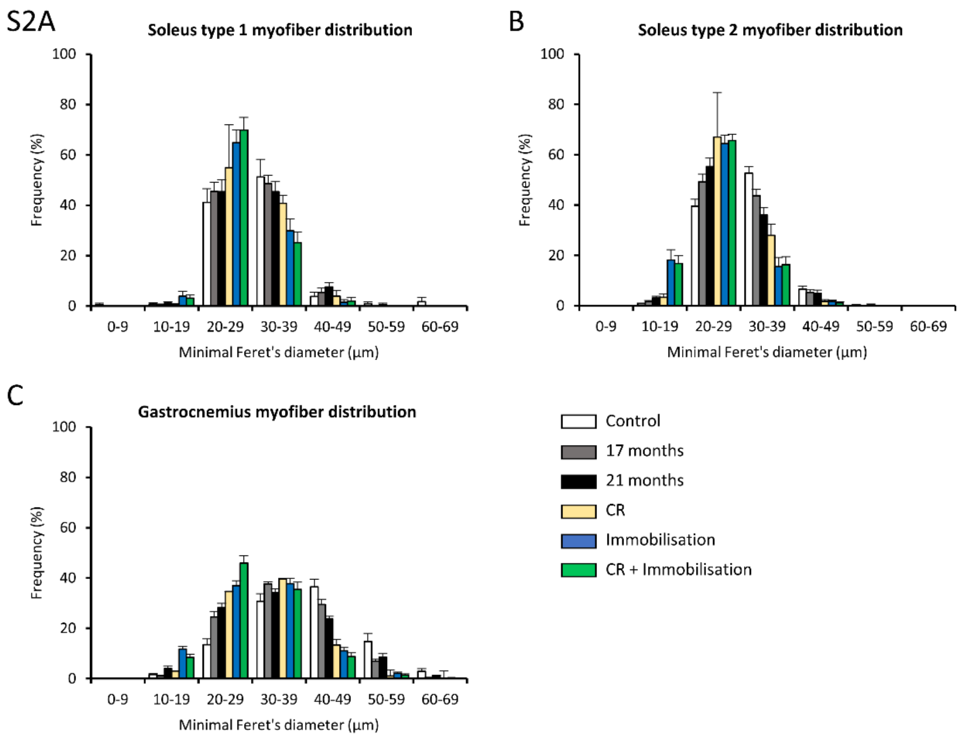

Supplementary Figure 2. (A-C) Myofiber size distribution based on their minimal Feret's diameter.

© 2022. de Jong J. et al. Published online at http://www.aginganddisease.org/EN/10.14336/AD.2022.1201

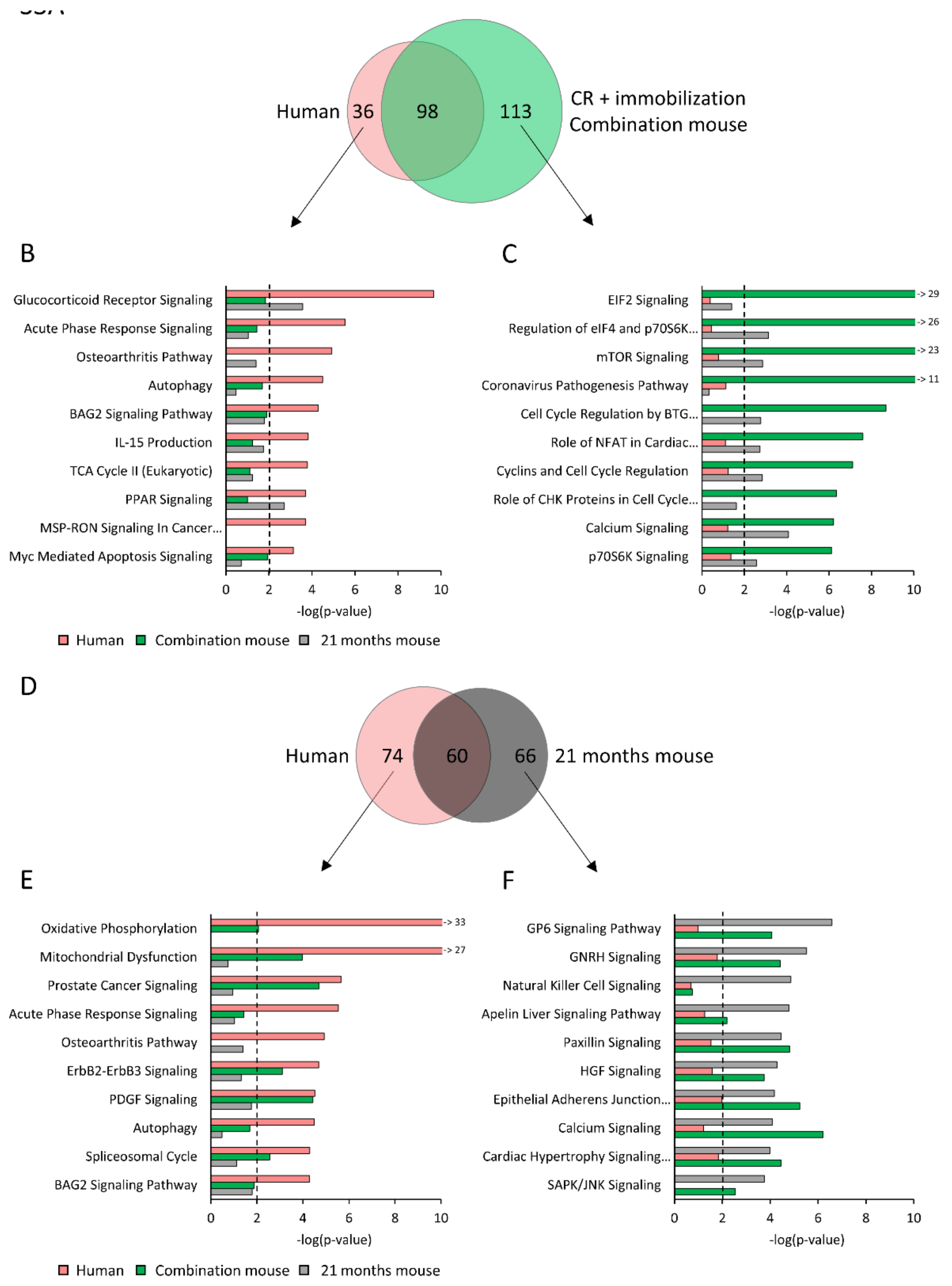

Supplementary Figure 3. (A) Venn-diagram displaying the number of differentially expressed pathways that are and are not shared by humans and the combination mouse model. (B) Top 10 DEPs found in humans that were not recapitulated by the combination mouse model. (C) Top 10 DEPs of combination model that did not overlap with old vs. young humans. Arrows with numbers indicate -log(p-value) of pathways with -log(p-value) greater than 10. (D) Venn-diagram displaying the number of DEPs that are and are not shared by humans and the aged mouse model. (E) Top 10 DEPs that were not recapitulated by the 21 months old mouse model. Arrows with numbers indicate -log(p-value) of pathways with -log(p-value) greater than 10. (F) Top 10 DEPs of 21 months old mice that did not overlap with old vs. young humans.

© 2022. de Jong J. et al. Published online at http://www.aginganddisease.org/EN/10.14336/AD.2022.1201

SUPPLEMENTARY DATA

Supplementary materials. List of differentially expressed pathways (old vs. young humans) per category:

Cell growth and proliferation
Glucocorticoid Receptor Signaling
Prostate Cancer Signaling
Pancreatic Adenocarcinoma Signaling
HER-2 Signaling in Breast Cancer
Chronic Myeloid Leukemia Signaling
Protein Kinase A Signaling
ErbB2-ErbB3 Signaling
BEX2 Signaling Pathway PDGF Signaling
Autophagy
AMPK Signaling
BAG2 Signaling Pathway
PTEN Signaling
JAK/Stat Signaling
IGF-1 Signaling
Apoptosis Signaling
Insulin Receptor Signaling T
ight Junction Signaling
Unfolded protein response
HIF1Î± Signaling
Adipogenesis pathway
Estrogen Receptor Signaling
BMP signaling pathway
Xenobiotic Metabolism AHR Signaling Pathway
Molecular Mechanisms of Cancer
Protein Ubiquitination Pathway
Glioma Signaling
PI3K/AKT Signaling
Non-Small Cell Lung Cancer Signaling
Myc Mediated Apoptosis Signaling
Hereditary Breast Cancer Signaling
Inhibition of ARE-Mediated mRNA Degradation Pathway
RAR Activation Senescence Pathway ERK/MAPK Signaling
Small Cell Lung Cancer Signaling
Thyroid Cancer Signaling
Role of JAK2 in Hormone-like Cytokine Signaling
STAT3 Pathway
FLT3 Signaling in Hematopoietic Progenitor Cells
ILK Signaling Androgen Signaling
Sumoylation Pathway
ERK5 Signaling Ceramide Signaling
14-3-3-mediated Signaling
IL-15 Signaling
Role of JAK1 and JAK3 in Î³c Cytokine Signaling
Death Receptor Signaling
Induction of Apoptosis by HIV1
Hypoxia Signaling in the Cardiovascular System
ATM Signaling
MIF-mediated Glucocorticoid Regulation
Estrogen-Dependent Breast Cancer Signaling
Ferroptosis Signaling Pathway
NAD Signaling Pathway
LPS-stimulated MAPK Signaling

© 2022. de Jong J. et al. Published online at http://www.aginganddisease.org/EN/10.14336/AD.2022.1201

SUPPLEMENTARY DATA

HIPPO signaling
FAT10 Cancer Signaling Pathway
Cell Cycle: G1/S Checkpoint Regulation
Endometrial Cancer Signaling
SPINK1 General Cancer Pathway
FAT10 Signaling Pathway
Cell Cycle Control of Chromosomal Replication
Necroptosis Signaling Pathway
ErbB Signaling

(mitochondrial) metabolism
Oxidative Phosphorylation
Mitochondrial Dysfunction
Sirtuin Signaling Pathway
Estrogen Receptor Signaling
Protein Kinase A Signaling AMPK Signaling
Insulin Receptor Signaling
TCA Cycle II (Eukaryotic)
PPAR Signaling
HIF1Î± Signaling
Xenobiotic Metabolism AHR Signaling Pathway
Gluconeogenesis I
PI3K/AKT Signaling
Glycolysis I
PPARÎ±/RXRÎ± Activation
IL-15 Signaling
Type II Diabetes Mellitus Signaling
Hypoxia Signaling in the Cardiovascular System
NAD Signaling Pathway

Inflammation
Glucocorticoid Receptor Signaling
Acute Phase Response Signaling
Chronic Myeloid Leukemia Signaling
Osteoarthritis Pathway
IL-15 Production
MSP-RON Signaling In Cancer Cells Pathway
PI3K Signaling in B Lymphocytes
IL-6 Signaling
Rac Signaling
PPARÎ±/RXRÎ± Activation
B Cell Receptor Signaling
Ceramide Signaling
IL-15 Signaling
IL-9 Signaling
Induction of Apoptosis by HIV1
MIF-mediated Glucocorticoid Regulation
Complement System
LPS-stimulated MAPK Signaling
Production of Nitric Oxide and Reactive Oxygen Species in Macrophages
IL-23 Signaling Pathway
4-1BB Signaling in T Lymphocytes
Oncostatin M Signaling
fMLP Signaling in Neutrophils

© 2022. de Jong J. et al. Published online at http://www.aginganddisease.org/EN/10.14336/AD.2022.1201

SUPPLEMENTARY DATA

Role of MAPK Signaling in Promoting the Pathogenesis of Influenza
IL-3 Signaling
MIF Regulation of Innate Immunity

Extracellular matrix
Osteoarthritis Pathway
Hepatic Fibrosis / Hepatic Stellate Cell Activation
Remodeling of Epithelial Adherens Junctions
Ephrin Receptor Signaling
Hepatic Fibrosis Signaling Pathway
Reelin Signaling in Neurons
Integrin Signaling
Agrin Interactions at Neuromuscular Junction
ILK Signaling
Estrogen Receptor Signaling
Tumor Microenvironment Pathway
Regulation Of The Epithelial Mesenchymal Transition By Growth Factors Pathway
Oncostatin M Signaling
Germ Cell-Sertoli Cell Junction Signaling

Neurology
Agrin Interactions at Neuromuscular Junction
Huntington's Disease Signaling
BAG2 Signaling Pathway
Ephrin Receptor Signaling
Neuregulin Signaling
Reelin Signaling in Neurons
NGF Signaling
Acute Myeloid Leukemia Signaling
Synaptogenesis Signaling Pathway
Axonal Guidance Signaling
GDNF Family Ligand-Receptor Interactions
Neurotrophin/TRK Signaling

Oxidative stress
Production of Nitric Oxide and Reactive Oxygen
Species in Macrophages
NRF2-mediated Oxidative Stress Response

© 2022. de Jong J. et al. Published online at http://www.aginganddisease.org/EN/10.14336/AD.2022.1201
